# Machine learning-based molecular detection using dark-field observation of two different nanoparticles

**DOI:** 10.1039/d6ra03733j

**Published:** 2026-07-31

**Authors:** Yuki Yano, Gen Hirao, Ryosuke Izumi, Yu Muto, Misato Yasuoka, Tsuyoshi Asahi, Takuo Tanaka, Mizuo Maeda, Tamotsu Zako

**Affiliations:** a Department of Chemistry and Biology, Graduate School of Science and Engineering, Ehime University 2-5 Bunkyo Matsuyama Ehime 790-8577 Japan zako.tamotsu.us@ehime-u.ac.jp; b Tokyo Research Centre, TOSOH Corporation 2743-1 Hayakawa Ayase Kanagawa 252-1123 Japan; c Department of Chemistry, Faculty of Science, Ehime University 2-5 Bunkyo Matsuyama Ehime 790-8577 Japan; d RIKEN Center for Advanced Photonics 2-1 Hirosawa Wako Saitama 351-0198 Japan; e RIKEN Pioneering Research Institute 2-1 Hirosawa Wako Saitama 351-0198 Japan

## Abstract

Metal nanoparticles, such as gold nanoparticles (AuNPs), are widely used as biosensing materials. In previous studies, dark-field microscopy (DFM) has been utilised to examine target-induced AuNP aggregation for molecular sensing. The intensity of scattering light of each spot observed by DFM was analysed at the single-cluster level for sensitive molecular detection. However, changes in the intensity and colour of AuNP aggregates were not significant when the inter-particle distance was large because of the insufficient effect of the surface plasmon resonance, suggesting difficulty in the sensitive detection of large molecules such as proteins. In this study, we developed a machine learning-based method to distinguish target-induced dimers from monomers by DFM, given large inter-particle distance, using two types of nanoparticles with different spot colours. When the two types of nanoparticles form a dimer (heterodimer), observation of a new spot colour derived from the heterodimer could be expected. As a proof-of-concept study, Protein A-modified silver nanoparticles and BSA-modified gold nanourchins were used to detect anti-BSA antibody; in the presence of the target, heterodimer was formed. The colours of individual spots observed by DFM at the single-cluster level were utilised for machine learning-based classification, and spots derived from heterodimers were identified for molecular detection. Our results demonstrate that the heterodimer formation increased in a target concentration-dependent manner. Furthermore, scattered lights from non-specific aggregates and impurities such as dust can be discriminated by this method. This assay is expected to be applicable to the detection of large molecules, such as proteins.

## Abstract 2

We developed a method to detect a target inducing heterodimer formation of heterogeneous nanoparticles using dark-field microscopy. Heterodimer was identified by classification of observed spots at the single cluster level using machine learning.

## Graphical abstract

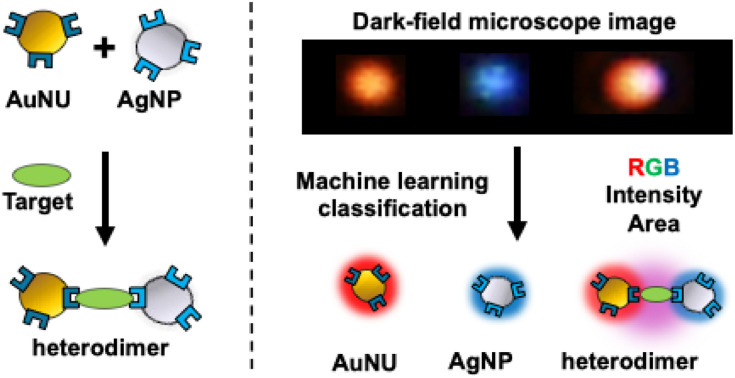

## Introduction

When nanometre-order metal nanoparticles are present as colloids within a solution, their optical properties vary in response to the size and shape of the nanoparticles.^1–4^ These properties have been utilised in various bio-applications.^5–7^ Because the change in the colour of such solutions is visible, they have frequently been employed as target detection indicators for disease markers, environmental contaminants, viruses, metal ions, and others.^8–12^ These detection mechanisms are often based on the formation of nanoparticle aggregates in the presence of target molecules.^13,14^ For these purposes, 15–40 nm gold nanoparticles (AuNPs) are primarily used. The AuNP solution exhibits a distinct colour change from red to blue when the aggregates are formed, indicating that the aggregates have absorption and scattering spectra different from those of the monomers. The use of AuNPs designed to interact specifically to the target enables target detection based on changes in optical properties.^8–10^

Dark field microscope (DFM) can be used to detect the aggregates by imaging scattered light from AuNPs as bright spots.^15–17^ As the spot colour differs according to particle size and shape, DFM can evaluate aggregate formation at the single-cluster level. We have developed a method using DFM to further increase the sensitivity of target detection based on AuNP aggregation.^18,19^ The versatility of target detection by DFM was demonstrated by evaluating the aggregates of AuNPs when the colour of a solution could not be accurately evaluated owing to contamination^20^ or when the concentration of target molecules was too low.^16^ Also, by using different spot colours of the AuNP monomer and aggregates, these could be discriminated by digital colour analysis of the whole DFM images^20–22^ or at the single-cluster level.^23,24^ In addition, it has been shown that machine learning can be used to identify spot colour change of AuNPs for the evaluation of salt-induced aggregation or the monitoring of catalytic reactions.^25,26^ Especially, Bennett *et al.* reported that identification of spot colour change of AuNPs using machine learning allows for the distinction of target-induced dimer from large aggregate, thereby enabling the detection of small molecules such as RNA.^27^ However, these analyses can only be applied when AuNPs form aggregates densely enough to affect the surface plasmon resonance. For example, Reinhard *et al.* reported that when AuNPs were aggregated by 40 base pairs (approximately 10 nm) of double-stranded DNA, no significant differences were observed in the spots' colour and intensity.^28^ Therefore, it is difficult to build assay systems using protein ligands such as antibodies or bulky targets such as viruses because the distances between particles are large.

**Scheme 1:**
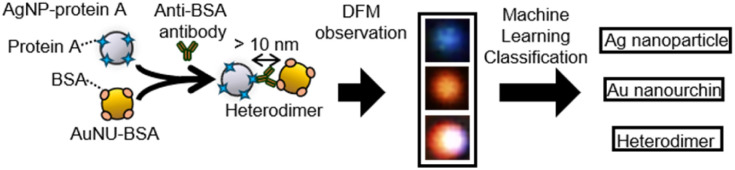
Schematic illustration of machine learning-based anti-BSA antibody detection using protein-modified silver nanoparticles and gold nanourchins.

In this study, with the aim of detection of large molecules such as proteins, we developed a machine learning-based method to distinguish target-induced heterodimers formed by two different metal nanoparticles from each nanoparticle, aggregate and noise in DFM images, utilising their spot colour, intensity and area as multiple information (Scheme 1). When the two types of nanoparticles form a dimer (heterodimer), observation of a new spot colour derived from the heterodimer could be expected. To distinguish heterodimer from monomer, 80 nm gold nanourchins (AuNUs) and 60 nm silver nanoparticles (AgNPs) with different scattering colour were used. AuNUs and AgNPs are observed as red colour spots and blue colour spots in the DFM images, respectively. In contrast, the spot colour of heterodimer is expected to be observed as a pink spot with a mixture colour of red and blue. Accordingly, we propose a novel assay system to observe the spots of the heterodimer formed in the presence of target molecules. As a proof-of-concept study, Protein A-modified AgNPs (AgNP-ProA) and BSA-modified AuNUs (AuNU-BSA) were used to detect anti-BSA antibody; in the presence of the target, heterodimer of AgNP-ProA and AuNU-BSA was formed. Each spot observed by DFM at the single-cluster level was classified using machine learning, and spots derived from heterodimers were identified for molecular detection. The RGB values, intensities and area of observed spots were used as the input data. These information make it possible to distinguish target-induced heterodimers from not only AuNU and AgNP, but also non-specific aggregate and noise. Our results demonstrated that the formation of heterodimers increased in a concentration-dependent manner of anti-BSA antibody, and 0.5 µg mL^−1^ (3.4 nM) anti-BSA antibody can be detected, suggesting a potential application towards the detection of large molecules such as proteins.

## Experimental

### Materials

80 nm AuNU and 60 nm AgNP were purchased from Cytodiagnostics (Burlington, ON, Canada). Bovine serum albumin (BSA), Protein A and polyethylene glycol 20 000 were purchased from FUJIFILM Wako Pure Chemicals (Osaka, Japan). Anti-BSA antibody and anti-Insulin antibody were purchased from Thermo Fisher Scientific (Waltham, MA, USA) and Santa Cruz Biotechnology Inc. (Dallas, TX, USA), respectively. Silane-coated glass slides and cover glass were purchased from Muto Pure Chemicals (Tokyo, Japan). Phosphate Buffer Saline (PBS) was purchased from Takara Bio Inc. (Shiga, Japan). Aqueous solutions were prepared using deionized Milli-Q water (Merck Millipore, Burlington, MA, USA).

### Synthesis of BSA-modified AuNUs and Protein A-modified AgNPs

Protein-modified nanoparticles were synthesised as previously described.^18,29^ Each AuNU and AgNP solution (500 µL) was centrifuged at 4500 rpm for 10 min, the supernatant was removed and the samples were redispersed in 100 µL of 0.1 × PBS. For the surface modification, 25 µg mL^−1^ of BSA and 10 µg mL^−1^ Protein A were added to the AuNU and AgNP solutions, respectively, followed by incubation at 37 °C for 30 min. Then 0.02% PEG was added to the mixed solution, and the unbound proteins were removed by centrifugation (4500 rpm (AuNU) and 10 000 rpm (AgNP) for 10 min), and 100 µL of 0.1× PBS was added to make stock solutions. The size of protein-modified nanoparticles was evaluated by dynamic light scattering (DLS) using Zetasizer-Nano ZS (Malvern Worcestershire, UK).

### TEM observation

Transmission electron microscopy (TEM) observation was performed in order to confirm the formation of heterodimer by addition of target anti-BSA antibody. 10 µL sample was spotted on collodion-film coated TEM copper grids and allowed to adsorb. Distilled water was added and excess suspension was removed from the grid using filter paper. The grids were air-dried prior to analysis. Samples were examined with an excitation voltage of 80 kV using a JEM-1400Plus TEM (JEOL, Tokyo, Japan).

### Observation of nanoparticles by dark field microscopy

Anti-BSA antibody (5 µL) at various concentrations (0–30 µg mL^−1^) was added to a mixed solution of 1.5 pM AuNU-BSA (5 µL) and 0.075 OD AgNP-ProA (5 µL) and incubated at 37 °C for 1 hour. These concentrations were determined as ones corresponding to equivalent number of particles. Anti-insulin antibody (5 µL, 30 µg mL^−1^) was used as a negative control target sample. After incubation, the mixed solutions including 0.5 pM nanoparticles and the target antibody (0–10 µg mL^−1^) were spotted on silane-coated glass slides and observed by DFM. This nanoparticle concentration was determined to minimise the possibility of individual nanoparticles being co-localised or in close proximity within a single spot. The images of bright spots obtained by DFM using BX53 microscope (Olympus, Tokyo, Japan) equipped with UDCW dark field condenser, UPlanFLN 60× objective and DP73 CCD camera as previously described,^19^ using the following parameters (ISO, 200; exposure time 20 ms; illumination intensity; maximum).

The spots were extracted with the ImageJ software using the following parameters (bright spot extraction threshold, 65–255; circularity, 0.3–1.0; size, 30–3000). The colour of each spot was separated into red (R), green (G) and blue (B) components, and the intensity and the size (area) of each component were also obtained using ImageJ.

### Machine learning

The training dataset was generated using spot information in the DFM images of the independently prepared samples of AuNU-BSA and AgNP-ProA in the absence or presence of anti-BSA antibody. Individual spots were randomly extracted and manually labelled into six classes; (1) AuNU-BSA (AuNU), (2) AgNP-ProA (AgNP), (3) heterodimer, (4) homodimer and (5) homoaggregate. For heterodimers, spots showing mixed colours of AgNP and AuNU or ones including both colours were extracted. For practical reasons, homodimer and homoaggregate were made since homodimers and homoaggregate were formed as non-specific aggregates mainly from AuNU-BSA. Spots were also observed in the DFM images when 0.1 × PBS containing no nanoparticles was used. Thus, training data of such spots was constructed as (6) noise. A total of 1607 labelled spots (AuNU, 304; AgNP, 261; heterodimer, 327; homodimer, 217; homoaggregate, 250; noise, 248) were used for model training and validation. Classification by machine learning was performed using R software (RStudio, ver. 4.3.0) including Karnlab package (for SVM model) and randomForest package (ver. 4.7-1.1) (for RF model). The code (for RF model) was shown in SI.

### Non-machine learning analysis

For comparison with the machine learning-based approach, a non-machine learning analysis was performed using the scattering intensity and area of the spots obtained from DFM images. The heterodimer ratio values were obtained using the histograms of intensity and area information from the DFM images at various target concentrations. To ensure a consistent baseline for comparison, the threshold for identifying heterodimers was determined so that the ratio at the negative control concentration (0 µg mL^−1^) matched the value obtained by the machine learning method (0.015). The proportion of spots exceeding this predefined threshold was then defined as the heterodimer ratio.

## Results and discussion

### Preparation of protein-modified nanoparticles and datasets for machine learning

To create heterodimers that maintain interparticle distances of 10 nm or greater, we used a complex of bovine serum albumin (BSA), Protein A, and anti-BSA antibody as a proof-of concept study (Scheme 1). Protein A and BSA were modified to purified AgNPs by density gradient centrifugation (AgNP-ProA) and AuNUs (AuNU-BSA) *via* physical adsorption, respectively. Usage of density gradient centrifugation enables AgNPs to unify blue colour (SI, Fig. S1). It was shown that the sizes of AgNP and AuNU were increased by 4 nm and 6 nm by modification, respectively (SI, Fig. S2A). By adding an anti-BSA antibody, complexes between different particles were formed, and spot colours were observed using DFM. Considering the size of antibody (approximately 8 × 14 nm)^30^ and the modified protein, the distance between AuNU and AgNP within their complex is estimated to exceed 18 nm. Both AuNU-BSA and AgNP-ProA maintained their sizes for 7 days when stored in 0.1× PBS at 4 °C, supporting stability of these nanoparticles at least for 1 week (SI, Fig. S2B)

**Fig. 1:**
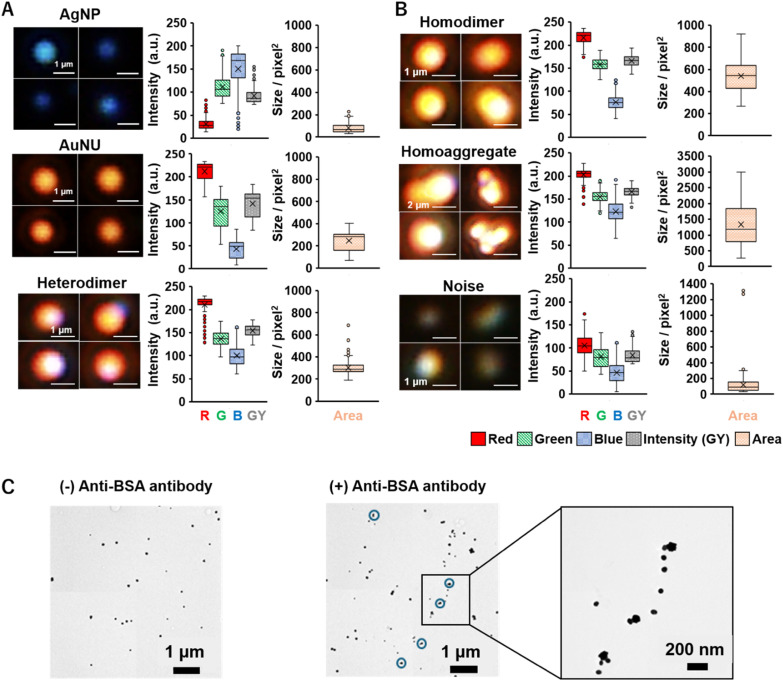
(A) DFM images of AgNP, AuNU and heterodimer spots (left) and their corresponding RGB, intensity and area distributions (right). (B) DFM images of homodimer, homoaggregate and noise spots (left) and their corresponding RGB, intensity and area distributions (right). (C) TEM images of AgNP-ProA and AuNU-BSA mixture samples in the absence (left) and presence of anti-BSA antibody (middle), where heterodimers are circled in blue. The right image shows a higher magnification image.

The observed spot colours were classified as AgNP, AuNU, heterodimer, homodimer, homoaggregate and noise using a machine learning-based system. First, we observed AgNP and AuNU by DFM, and confirmed that they exhibited distinguishable spot colours. Fig. 1A presents DFM images and corresponding RGB, intensity and area values of AgNP-ProA and AuNU-BSA samples, which were analysed by ImageJ software.^31,32^ The former primarily showed blue spots (Fig. 1A upper left), whereas the latter mainly showed red spots (Fig. 1A middle left). These differences in colour are also evident from the spots' RGB values (Fig. 1A upper right and middle right).

To form the heterodimer, anti-BSA antibodies were added to AgNP-ProA and AuNU-BSA samples, and the change in spot colour was observed using DFM. Pink-coloured spots with a mixture of red and blue were observed, which were absent in the only AgNP-ProA and AuNU-BSA samples, suggesting that these spots were derived from the heterodimers (Fig. 1A lower left). This is justified by a numerical study using a finite element method with empirical values for Au and Ag,^33^ showing that the absorbance spectra of AuNP and AgNP are not affected when the interparticle distance is longer than 5 nm (SI, Fig. S3). This result is also supported by the absorbance spectra of the solutions containing AgNP-ProA and AuNU-BSA in the absence and presence of anti-BSA, where no significant change in the spectra was observed in the presence of anti-BSA (SI, Fig. S4). It is noted that a slight red shift was observed in the presence of anti-BSA at high concentration, possibly due to non-specific aggregation.

Heterodimer formation was confirmed by transmission electron microscopy (TEM) in the presence and absence of anti-BSA antibody (Fig. 1C). In the former case, dimer formation was observed while no dimer was observed in the latter case. The particle shapes revealed that AgNP-ProA and AuNU-BSA were also present as dimers, suggesting that the pink spots observed during dark-field observation were identified as heterodimers. The histograms of nanoparticle size obtained from the TEM images in the absence or the presence of anti-BSA antibody support the heterodimer formation; the nanoparticle assemblies sizing from 120 to 160 nm increased in the presence of anti-BSA antibody, while the sizes of AuNU-BSA and AgNP-ProA were 91.7 and 53.9, respectively (SI, Fig. S5).

For training data for machine learning, spots corresponding (1) AgNP, (2) AuNU and (3) heterodimer were extracted (Fig. 1A). For practical reasons, training data of non-specific aggregates such as (4) homodimer, (5) homoaggregate were also made (Fig. 1B).

Importantly, it is necessary to exclude any spots originating from impurities as a noise. Thus, training data of spots observed in the solution containing no nanoparticles was constructed as (6) noise (Fig. 1B). Unfocused spots were also classified here. Except for AgNP-ProA, AuNU-BSA and heterodimer, we analysed RGB values, intensity and area of homodimer, homoaggregate and noise. It is shown that the size of heterodimer is different from that of homodimer and homoaggregate (Fig. 1A and B). It was thus expected that the parameters such as RGB values, intensity and area of heterodimer, homodimer and homoaggregates would be distinguishable from those of AgNP and AuNU by machine learning.

### Anti-BSA antibody detection using machine learning classification

The R software was used to analyse the data for the construction of the classifier system for the spots. As a machine learning algorithm, we used Random Forest (RF), suitable for data classification.^34^ Data classification using RF allows the attribution of data with unknown attributes based on the training data. It has been reported that it is possible to classify even multivariate and nonlinear classification problems with excellent accuracy. Using each spot as a variable, we constructed an RF-based discrimination system for spot attribution using RGB, intensity and area values. The classification performance of proposed system was evaluated by cross-validation using macro-*F*_1_ score (SI, Table S1). For comparison, classification using other colour spaces, such as HSV (hue, saturation and value) and LAB (lightness, a* vector and b* vector) was examined using training datasets including intensity and area information (SI, Table S2). The result indicates that all three colour spaces showed similar classification performance, suggesting that the classification performance does not depend on the choice of colour information. It is noted that the classification performance decreased for all the three colour spaces when intensity and area information were not used, supporting the importance of these parameters.

**Fig. 2:**
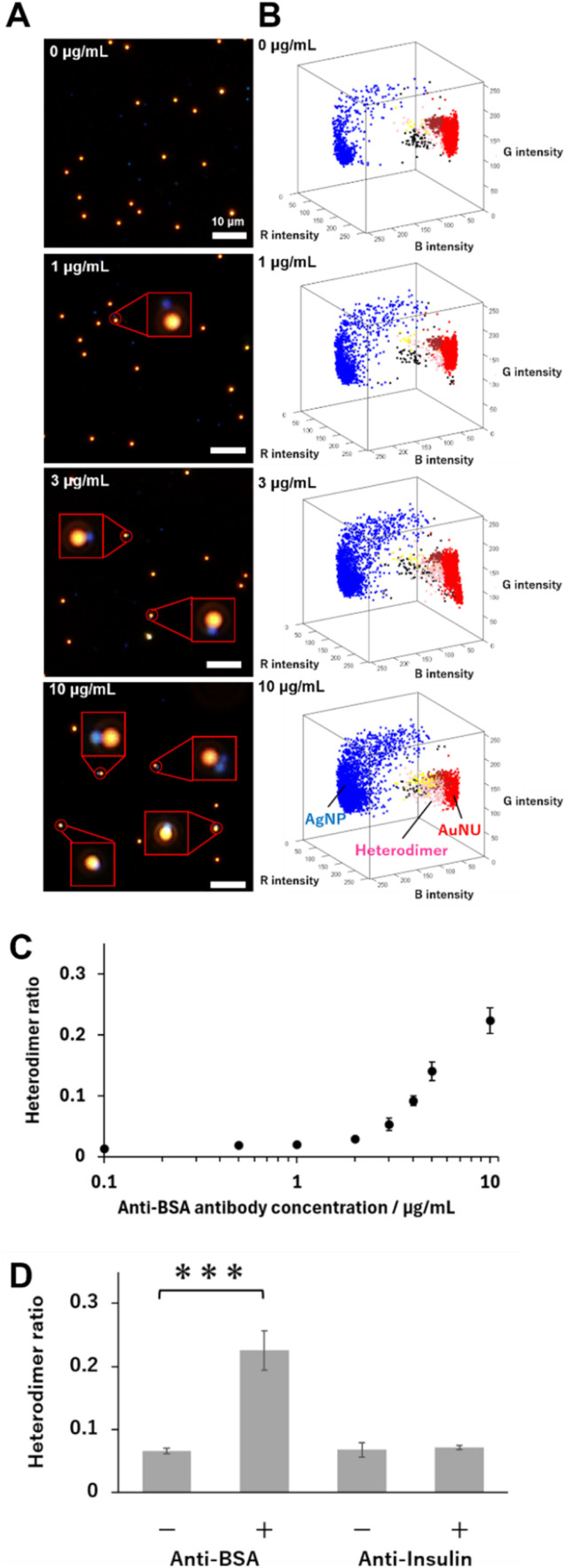
(A) DFM images of AuNU-BSA and AgNP-ProA mixture samples in the absence and presence of anti-BSA antibody. Red squares show higher magnification images of heterodimer. (B) RGB 3D distribution of each class: AgNP (blue), AuNU (red), heterodimer (pink), homodimer (brown), homoaggregate (yellow), noise (black). Concentrations of anti-BSA antibody are shown. (C) Anti-BSA antibody detection using heterodimer ratio based on spot classification by RF. (D) Evaluation of the specificity using anti-insulin antibody as a non-target protein. ***p-value < 0.005.

The heterodimer ratio was defined as the number of heterodimers divided by the total number of AuNUs, AgNPs, and heterodimers:
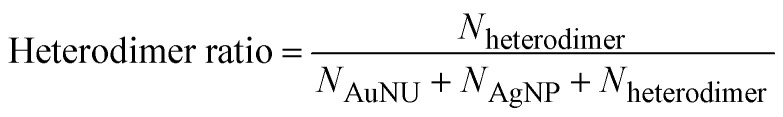


Then the constructed machine learning system was applied for the target molecule detection. Anti-BSA antibody at various concentrations were added to the mixture of the AgNP-ProA and AuNU-BSA samples. The DFM images of each sample were observed (Fig. 2A and SI, S6A) and analysed using the spot classification method. Fig. 2B and S6B shows that RGB 3D distribution of each class. The classified spots as heterodimer increased in a concentration-dependant manner and did not belong to neither AgNP nor AuNU, suggesting that the spots were heterodimer to own the characteristics of AgNP and AuNU.

Fig. 2C and Table 1 showed the ratio of heterodimer. As shown in the figure, the ratio increased in a concentration-dependent manner with the anti-BSA antibody, indicating that heterodimer formation is due to the target antibody. In addition, a significant difference from the blank was shown by *t*-test at 0.5 µg mL^−1^ (3.4 nM) and higher (SI, Fig. S7). Then the limit of detection (LOD) for this sensing system was determined to be 0.5 µg mL^−1^. It is noted that a trace amount of heterodimers, including possible assembly of two different homodimers, were formed even in the absence of target due to non-specific interaction between AuNUs and AgNPs. However, the ratio was 0.015 and negligible. Importantly, heterodimer was not formed when the AgNP-ProA and AuNU-BSA samples were incubated with anti-insulin antibody as a negative control while heterodimer was formed in the presence of anti-BSA antibody (Fig. 2D), supporting that heterodimer formation was induced by antigen–antibody interactions rather than non-specific interaction.

**Table 1:** Spot classification using RF algorithm

	AgNP-ProA	AuNU-BSA	Heterodimer	Heterodimer ratio
0 µg mL^−1^	715	4190	77	0.0152
1 µg mL^−1^	802	2532	76	0.0217
2 µg mL^−1^	497	2327	161	0.0300
10 µg mL^−1^	161	442	186	0.230

For comparison, support vector machine (SVM) algorithm, which is also suitable for supervised machine learning, was also used.^35,36^ The sensitivity using SVM classification was similar to that of RF (SI, Fig. S8 and Table S3), and approximately 10-times better than that of the previously reported method using BSA-modified AuNP which could observe only large aggregates by DFM (3.0 µg mL^−1^).^17^

### Comparison with other machine learning methods and non-machine learning methods

Other machine learning algorithms such as Linear Discriminant Analysis (LDA), Logistic Regression (LR), and Decision Trees (DT) were utilised for comparison. LDA derives a linear discriminant that maximises the ratio of between-group variance to within-group variance; unknown samples are subsequently classified into groups based on their scores from the derived function.^37^ LR is a statistical method that estimates the probability of a binary outcome occurring based on one or more explanatory variables.^38^ DT is a tree-structured model used for classification and regression, serving as the base learners for RF.^38^ LR achieved macro *F*_1_-score equivalent to that of RF while DT exhibited a lower value, with LDA yielding the lowest value (SI, Table S1). This result suggests that the variance reduction effect by ensemble learning methods such as RF could contribute to improved performance. Furthermore, although the heterodimer ratio increased in a concentration-dependent manner across all methods except for LDA (SI, Fig. S9), RF showed the most favourable performance in terms of LOD and classification performance.

**Fig. 3:**
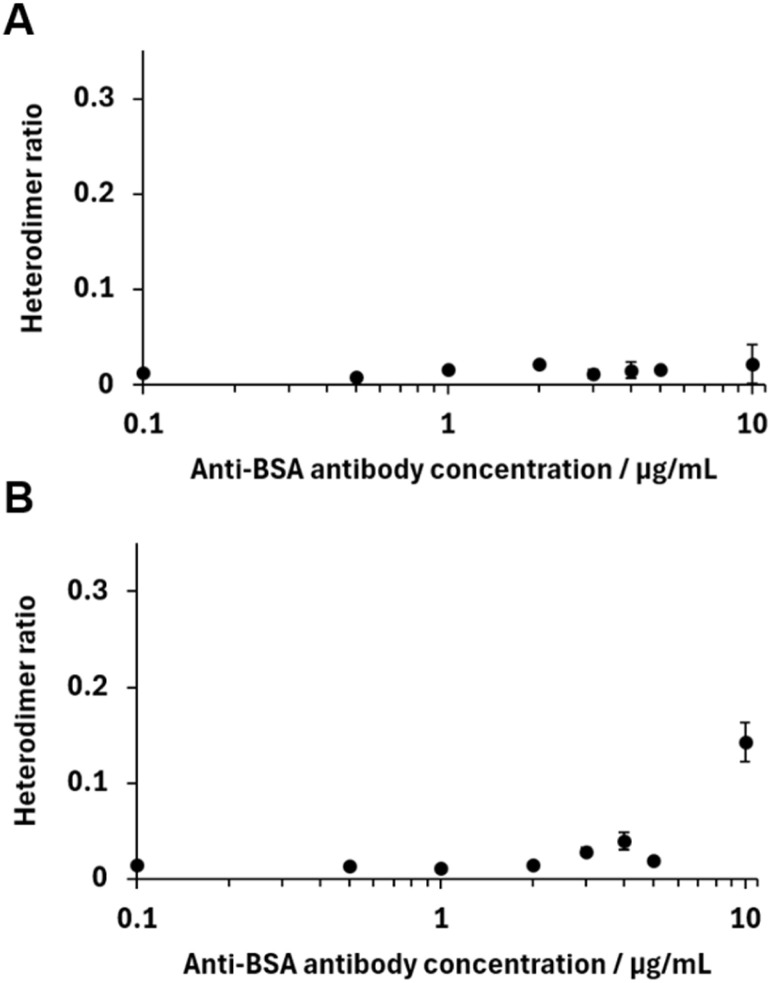
Heterodimer ratio using non-machine learning methods based on intensity (A) and area (B) information. The threshold for identifying heterodimers was determined such that the heterodimer ratio at the negative control concentration (0 µg mL^−1^) matched the value obtained by RF (0.015). The LOD values were N.D. (intensity) and 3 µg mL^−1^ (area), respectively (see Fig. S10 (SI)).

For the comparison with a non-machine learning method, the threshold values to distinguish heterodimer were set to match the heterodimer ratio without the target to 0.015 as obtained by RF (Table 1 and Fig. 2C). Consequently, the analysis using intensity showed no significant increase in the heterodimer ratio even at 10 µg mL^−1^ target anti-BSA antibody (Fig. 3A and SI, Fig. S10). In contrast, while the area-based analysis revealed a concentration-dependent increase, the LOD remained at 3 µg mL^−1^ (Fig. 3B and SI, Fig. S10), lower sensitivity compared to one obtained by the machine learning method (0.5 µg mL^−1^).

These results support capability of machine learning-based classification of nanoparticles in DFM images for the sensitive detection of anti-BSA. This sensitivity permits direct detection of target molecules based on scattering optical properties without the necessity of enzymatic amplification or fluorescent labelling. The sensitivity is also comparable to that of antibody detection methods such as ones using field-effect transistor (FET) based biosensor (0.62 µg mL^−1^),^39^ paper-based fluoroimmunoassay (5.0 µg mL^−1^),^40^ paper-based ELISA (9.0 µg mL^−1^),^41^ plate-based ELISA (3.0 µg mL^−1^).^42^ Therefore, the current approach could serve as a basis for future development of analytical methods for detecting target molecules.

For a practical application, impurities included in biological samples such as blood would affect the detection. For example, non-specific aggregates of nanoparticles could be formed even in the absence of the target molecule in biological samples. It is also possible that such impurities could cause noise spots. Thus, it would be important to enrich the training data of blank samples of biological samples in the absence and presence of nanoparticles. Furthermore, it has also been reported that fibrinogen present in blood readily adsorbs onto the surfaces of AuNPs,^43^ which may cause inhibition of binding to the target molecule. In such cases, modification of NPs surface with polyethylene glycol (PEG) following protein immobilization would be effective to inhibit further adsorption of other biological components.^44^ It is also possible that biological materials such as proteins could inhibit adsorption of NPs on the silane-coated glass slide. To resolve this issue, it is necessary to utilise a strong interactions such as biotin-streptavidin interaction to attach the nanoparticles to the glass slide.^45^

## Conclusion

In this study, we developed a method for detecting colour differences in spots of heterodimer formed by two different nanoparticles *via* DFM observation, even in dimers where the distances between particles are so large that surface plasmon resonance change does not occur. As a proof-of-concept study, two nanoparticles were modified with antigen and Protein A, respectively, and two mixed-colour spots were observed depending on target antibody concentration. The results suggest that a heterodimer was formed in the presence of the target and heterodimer formation could be observed as a colour difference from the colours of monomer particles. Furthermore, we developed a method to quantitatively evaluate heterodimers from observed images using a machine learning-based spot classification system. It is noted that scattered lights from nanoparticles and impurities such as dust can be discriminated by this method. It is also envisaged that the integration of detailed information from each pixel of spots would enable even more precise particle classification in future studies. This assay method is expected to be applied not only to the antigen and antibody used in this study, but also to the detection of large molecules such as proteins and virus particles.

## Author contributions

T. Z. conceived the project. T. Z., Y. Y., G. H., R. I., T. A. and M. M. designed the experiments. Y. Y., G. H., R. I. and Y. M. performed the experiments. T. T. performed the simulations. Y. Y., G. H., R. I., M. Y. analysed the data. Y. Y., G. H., R. I., Y. M. and T. Z. wrote the initial manuscript, and all the authors discussed the results and contributed to the manuscript preparation.

## Conflicts of interest

There are no conflicts to declare.

## Supplementary Material

RA-OLF-D6RA03733J-s001

## Data Availability

The data used in the current study are available from the corresponding author on reasonable request. The data supporting this article have been included as part of the supplementary information (SI). Supplementary information is available. See DOI: https://doi.org/10.1039/d6ra03733j.
