# Supplementary material for: Machine learning-based molecular detection using dark-field observation of two different nanoparticles
Source: RSC Adv. 2026 Jul 31. Online ahead of print. doi: 10.1039/d6ra03733j (PMC13426280; doi:10.1039/d6ra03733j)
Supplement: RA-OLF-D6RA03733J-s001 [file RA-OLF-D6RA03733J-s001.pdf]

Supplementary Information (SI) for RSC Advances.
This journal is © The Royal Society of Chemistry 2026

Machine learning-based molecular detection using dark-field observation of two different nanoparticles

Yuki Yano[a†], Gen Hirao [a†], Ryosuke Izumi[a†], Yu Muto[a,b], Misato Yasuoka[c], Tsuyoshi Asahi[a], Takuo Tanaka[d], Mizuo Maeda[e] and Tamotsu Zako*[a,c,d,e]

a. *Department of Chemistry and Biology, Graduate School of Science and Engineering, Ehime University, 2-5 Bunkyo, Matsuyama, Ehime 790-8577, Japan,* E-mail: *zako.tamotsu.us@ehime-u.ac.jp*
b. *Tokyo Research Centre, TOHSO Corporation, 2743-1 Hayakawa, Ayase, Kanagawa 252-1123, Japan.*
c. *Department of Chemistry, Faculty of Science, Ehime University, 2-5 Bunkyo, Ehime, Matsuyama, 790-8577, Japan.*
d. *RIKEN Center for Advanced Photonics, 2-1 Hirosawa, Wako, Saitama 351-0198, Japan.*
e. *RIKEN Pioneering Research Institute, 2-1 Hirosawa, Wako, Saitama 351-0198, Japan.*

[†] These authors contributed this work equally.
[*]E-mail: zako.tamotsu.us@ehime-u.ac.jp

Table of Contents

Materials and Methods
Figure S1 DFM images of unpurified and purified AgNPs and size evaluation of each AgNP
Figure S2 Size evaluation of AuNU, AuNU-BSA, AgNP, AgNP-Protein A, and stability of AuNU-BSA and AgNP-ProA
Figure S3 Simulation study of plasmon coupling effect of heterodimers
Figure S4 Absorbance spectrum of AuNU-BSA and AgNP-ProA in the absence and presence of anti-BSA antibody
Figure S5 Size evaluation of each particle obtained from TEM images
Table S1 Model evaluation of SVM, RF, LDA, LR and DT
Table S2 Classification performance using various colour spaces
Figure S6 DFM images of the samples incubated with anti-BSA antibody and RGB 3D plot distribution
Figure S7 Anti-BSA antibody detection using heterodimer ratio based on spot classification by RF
Figure S8 Anti-BSA antibody detection using heterodimer ratio based on spot classification by SVM
Table S3 Spot classification by SVM algorithm
Figure S9 Anti-BSA antibody detection using heterodimer ratio based on spot classification by other machine learning methods using LDA, LR and DT
Figure S10 Heterodimer ratio using non-machine learning methods based on intensity and area information

Methods

Purification of AgNPs by density gradient centrifugation

Sucrose was purchased from FUJIFILM Wako (Osaka, Japan). Since various colours of spots were observed for the commercial AgNPs by DFM (Fig. S1), AgNPs were purified by density gradient centrifugation on the basis of the study reported by Lee *et. al*[1].. In this study, first, sucrose concentration gradients (70, 60, 50, 40, 30 % from the bottom) were made in centrifuge tubes. Then 20-folds concentrated 60 nm AgNPs solution was added to the top of the centrifuge tube in which the sucrose concentration gradient was prepared, and the tube was centrifuged at 4000 rpm for 30 min. The upper and lower layers of the AgNP layer were carefully extracted, and the extracted AgNPs were spotted on glass slides and observed by DFM (Fig. S1A). The concentration of AgNP was determined using absorbance at 416 nm according to the manufacture's instruction using UV-Vis spectrophotometer (V-670, Jasco, Tokyo, Japan). The sizes of unpurified and purified AgNPs were evaluated by DLS using Zetasizer-Nano ZS (Malvern Worcestershire, UK) (Fig. S1B), indicating that relatively smaller size of AuNPs were obtained. Unpurified AgNPs were used for comparison. (Fig. S1A). The purified AgNP sample was dialysed with MQ water using a dialysis cup (Slide-A-Lyzer MINI Dialysis Device, 3.5K MWCO, Thermo Fisher Scientific).

Simulation study of plasmon coupling effect

The absorbance spectra shown in Figure S3 were obtained using the finite element method (FEM) software package COMSOL Multiphysics, with empirical value for the permittivity (e) of Au and Ag[2]. The radii of Au and Ag nanoparticles were 40 nm and 20 nm, respectively. Both nanoparticles were surrounded with water (n = 1.333). The gap distance between the AuNP and AgNP was varied from 1 to 20 nm in 1 nm increments.

Machine learning

The training dataset was generated from the DFM images of the independently prepared samples of AuNU-BSA and AgNP-ProA in the absence or presence of anti-BSA antibody. Individual spots were randomly extracted and manually labelled into six classes; 1) AuNU-BSA (AuNU), 2) AgNP-ProA (AgNP), 3) heterodimer, 4) homodimer and 5) homoaggregate. For heterodimers, spots showing mixed colours of AgNP and AuNU or ones including both colours were extracted. For practical reasons, homodimer and homoaggregate were made since homodimer and homoaggregate were formed as non-specific aggregates mainly from AuNU-BSA. Spots observed in the DFM images of 0.1 × PBS sample containing no nanoparticles were used to make the training data of 6) noise.

Classification by machine learning was performed using R software (RStudio, ver. 4.3.0) including Karnlab package (for SVM model) and randomForest package (ver. 4.7-1.1) (for RF model). The code (for RF model) was shown below.

```
install.packages("tcltk")
install.packages("randomForest")

RF_classifier_hetero <- function(testData_name = "RGB-Gray-Area.csv", trainData_name = "
trainingDataset.csv") {
  library(tcltk)
  library(randomForest)

  train <- read.csv(trainData_name)
  train$Class <- as.factor(train$Class)
```

```
RF_model <- randomForest(Class ~ ., data = train)

Data <- read.csv(testData_name)
predictData <- predict(RF_model, Data)

AuNU <- c()
AgNP <- c()
homodimer <- c()
heterodimer <- c()
agg <- c()
noise <- c()

pb <- txtProgressBar(min = 1, max = length(predictData), style = 3)

for (i in 1:length(predictData)) {
  if (predictData[i] == "AuNU") {
    AuNU <- c(AuNU, i)
  } else if (predictData[i] == "AgNP") {
    AgNP <- c(AgNP, i)
  } else if (predictData[i] == "homodimer") {
    homodimer <- c(homodimer, i)
  } else if (predictData[i] == "heterodimer") {
    heterodimer <- c(heterodimer, i)
  } else if (predictData[i] == "agg") {
    agg <- c(agg, i)
  } else if (predictData[i] == "noise") {
    noise <- c(noise, i)
  } else {
    print(paste("Unexpected prediction:", predictData[i]))
    stop("Error!!!")
  }

  setTxtProgressBar(pb, i)
}

close(pb)
```

References

1 S. H. Lee, B. K. Salunke and B. S. Kim, *Biotechnol. Bioprocess Eng.*, 2014, **19**, 169–174.
2 P. B. Johnson and R. W. Christy, *Phys. Rev. B*, 1972, **6**, 4370–4379.

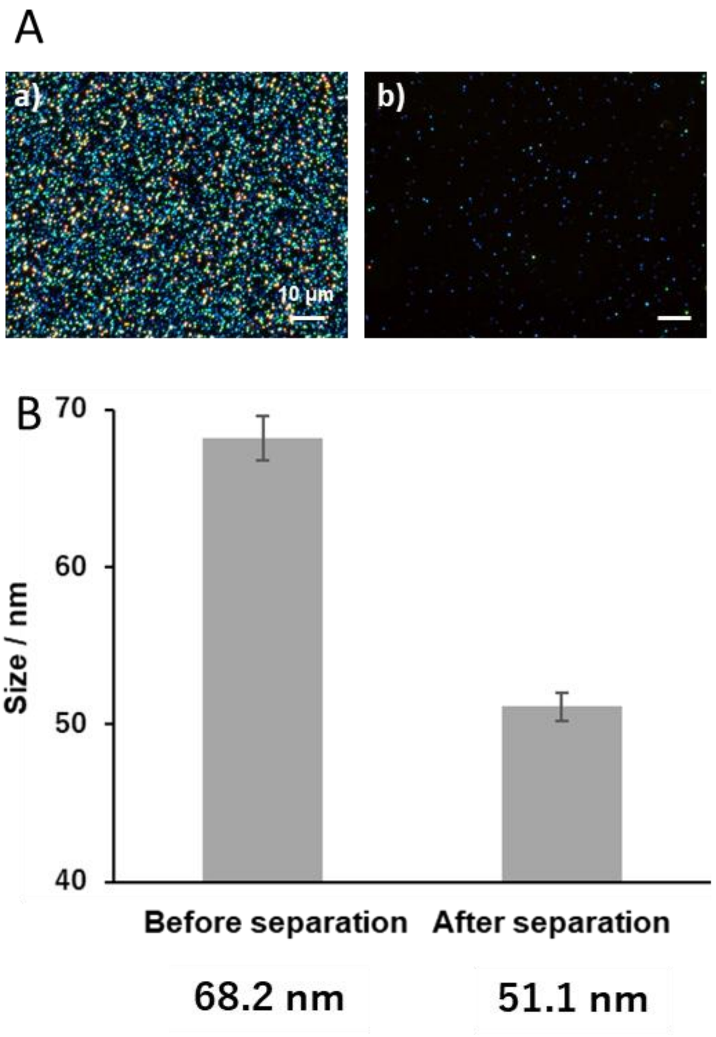

Figure S1 DFM images of unpurified and purified AgNPs and size evaluation of each AgNP. (A) DFM images of AgNPs before separation (left) and after separation (right). (B) Size evaluation of AgNPs before and after separation using DLS.

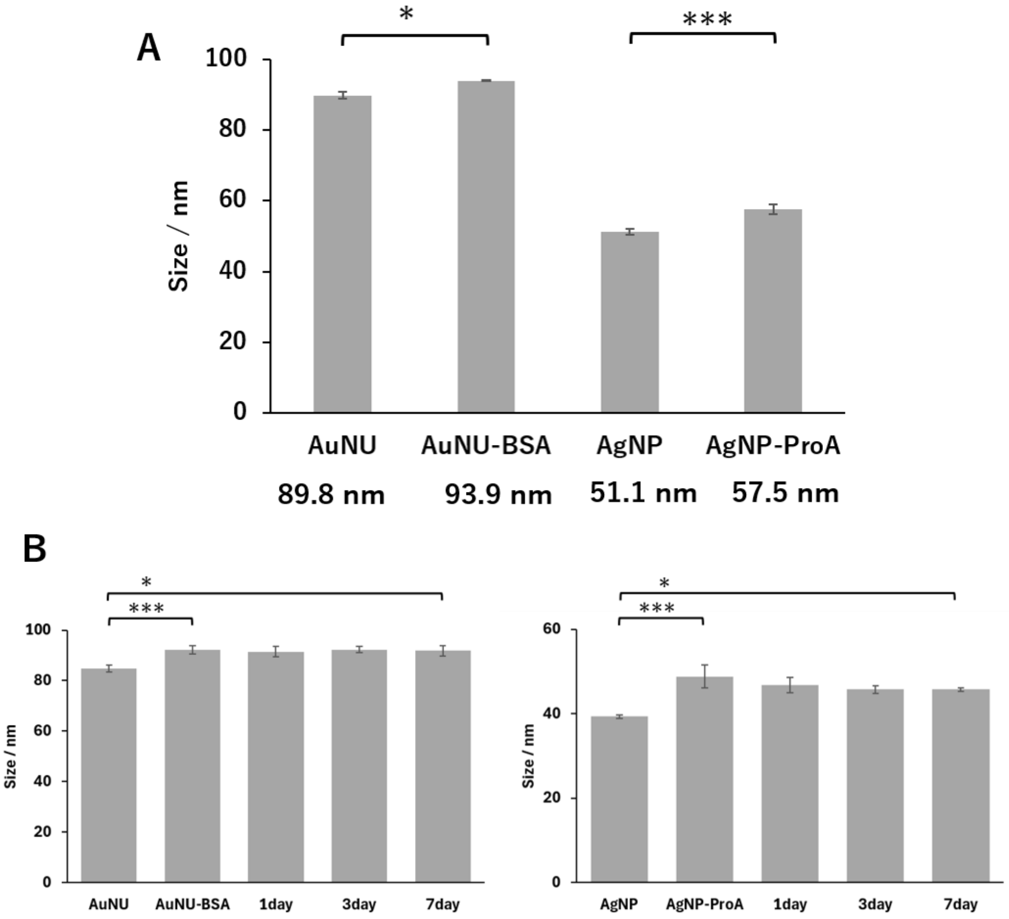

Figure S2 (A) Sizes of AuNU, AuNU-BSA, AgNP and AgNP-Protein A(AgNP-ProA) using DLS. (B) Stability of AuNU-BSA (left) and AgNP-ProA (right). AuNU-BSA and AgNP-ProA samples were stored in 0.1 × PBS at 4°C for indicated time period, and the size was measured using DLS. * p-value < 0.05, *** p-value < 0.005.

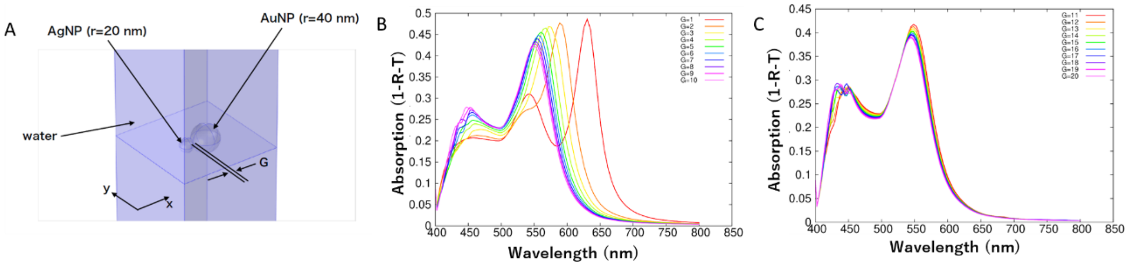

Figure S3 Simulation study of plasmon coupling effect of heterodimers. A) simulation condition of AuNP and AgNP. B) Simulated absorbance of heterodimer changing gap between AuNP and AgNP from 1 – 10 nm. C) Simulated absorbance of heterodimer changing gap between AuNP and AgNP from 10 – 20 nm.

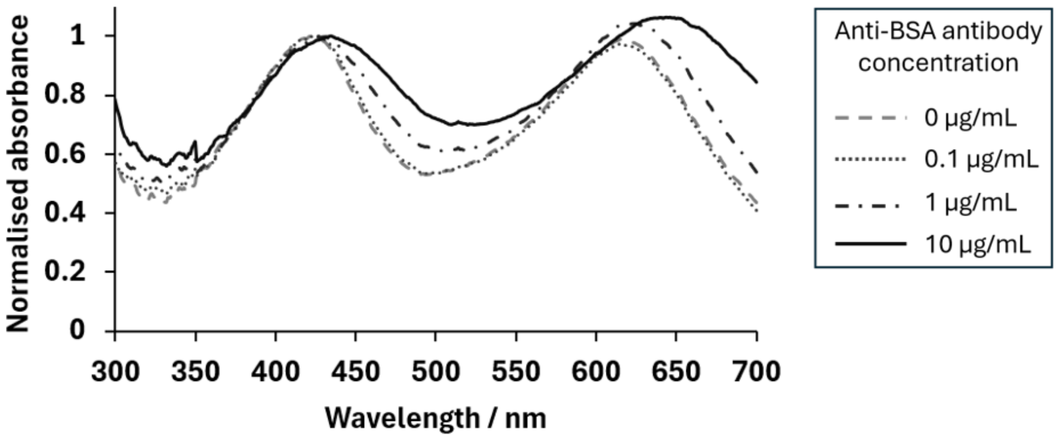

Figure S4 Absorbance spectra of AuNU-BSA and AgNP-ProA in the absence and presence of anti-BSA antibody, measured by UV-Vis spectrophotometer (V-670, Jasco, Tokyo, Japan).

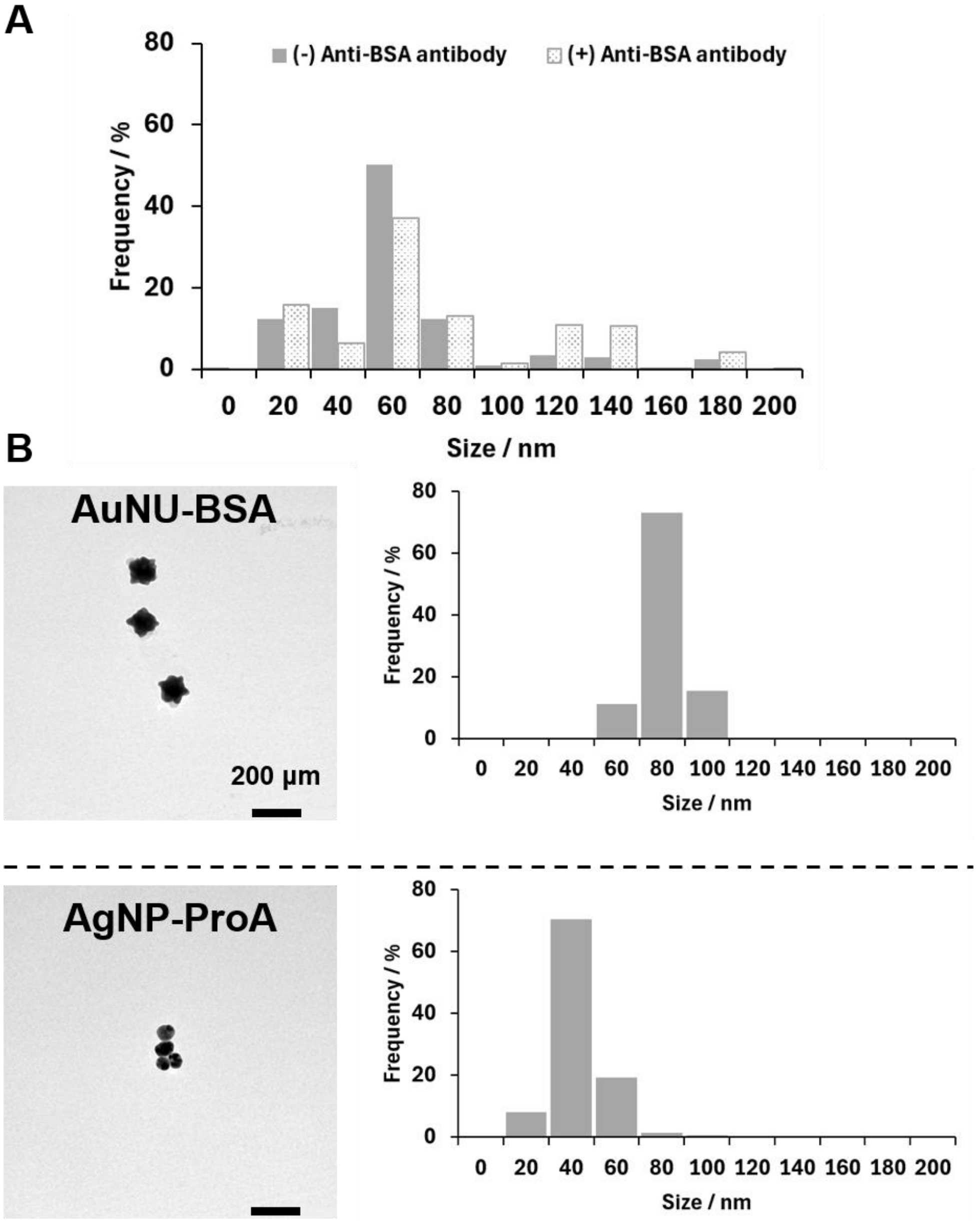

Figure S5 Size evaluation of each particle obtained from TEM images. A) Histogram of the particle size measured from TEM images in the absence and presence of anti-BSA antibody. B) TEM images of AuNU-BSA and AgNP-ProA (left) and histogram of obtained particle size (right). The average size of AuNU-BSA and AgNP-ProA were 91.7±9.7 nm and 53.9±11.68 nm, respectively.

Table S1 Model evaluation of Support Vector Machine (SVM), Random Forest (RF), Linear Discriminant Analysis (LDA), Logistic Regression (LR) and Decision Tree (DT)

	Macro-F1 Score	Standard Deviation
SVM	0.976	0.008371
RF	0.977	0.012371
LDA	0.897	0.1259
LR	0.973	0.008513
DT	0.94	0.010876

F1 score is a metric for evaluating classification performance, defined as the harmonic mean of precision and recall of classification. Macro-F1 scores of SVM, RF, LDA, LR and DT were obtained using stratified 10-fold cross-validation implemented in R. The dataset was divided into ten subsets while preserving the class distribution, and in each iteration, 90% of the data were used for training and 10% for testing.

Table S2 Classification performance using various colour spaces

Features	Macro-F1 Score	Standard Deviation
RGB+intensity+area	0.973	0.009
HSV+intensity+area	0.972	0.011
LAB+intensity+area	0.972	0.007
RGB only	0.880	0.031
HSV only	0.883	0.025
LAB only	0.886	0.021

RGB (Red, Green and Blue), intensity and area for each spot were obtained using ImageJ. HSV (Hue, Saturation, Value) and LAB (lightness, a^* vector, b^* vector) features were converted from RGB values using an Excel macro. All macro-F1 scores were obtained using a RF model implemented in R.

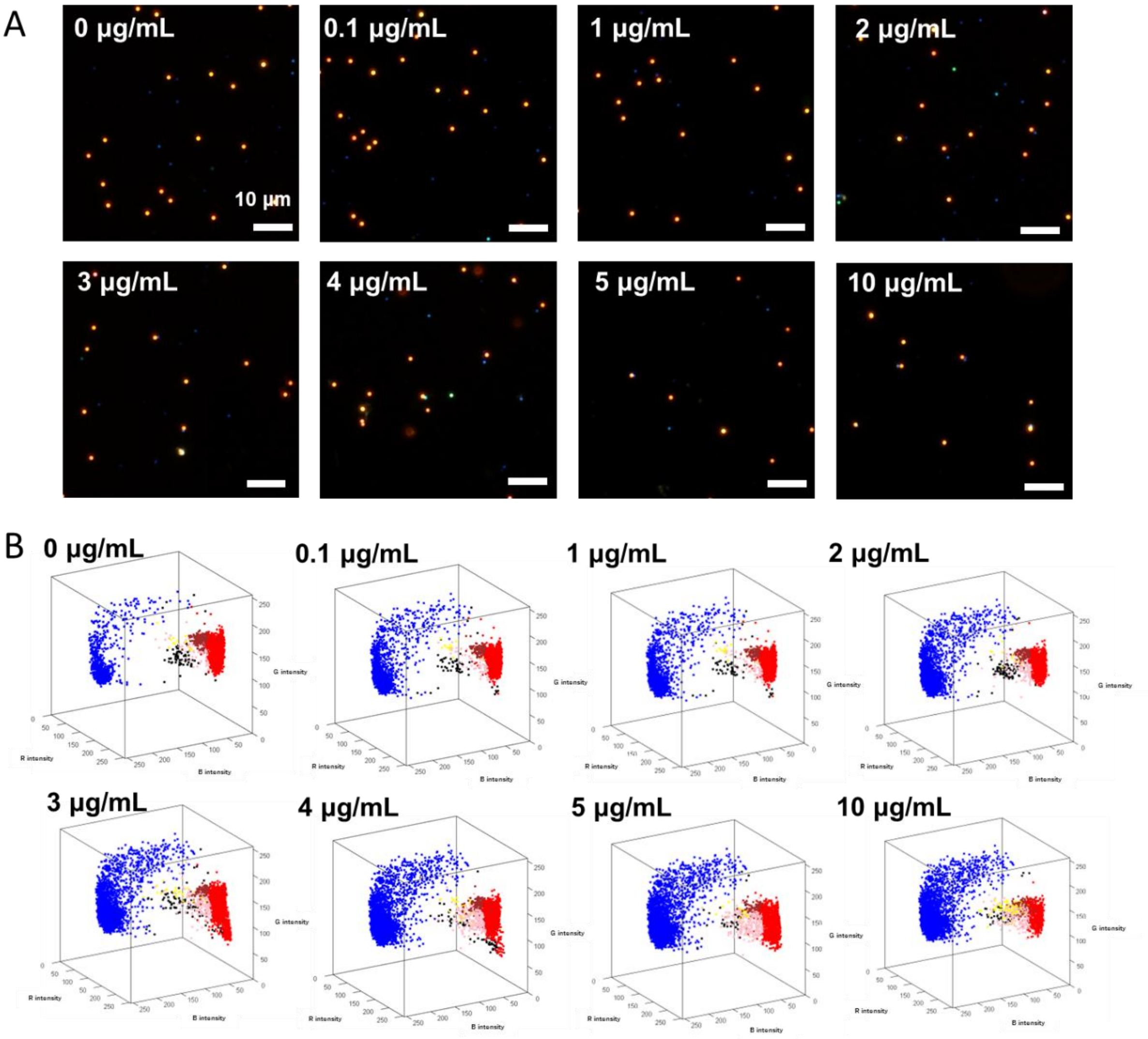

Figure S6 (A) DFM images of the samples incubated with anti-BSA antibody and (B) RGB 3D distribution of each class. Concentrations of anti-BSA antibody are shown.

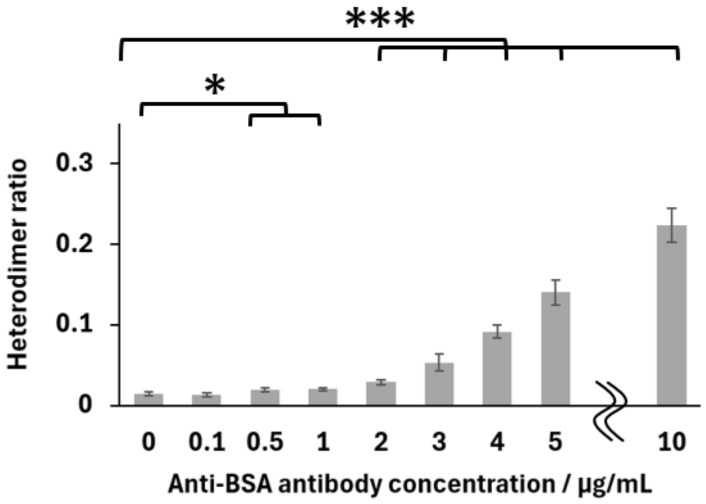

Figure S7 Anti-BSA antibody detection using heterodimer ratio based on spot classification by RF on a broken scale. * p-value < 0.05; *** p-value < 0.005

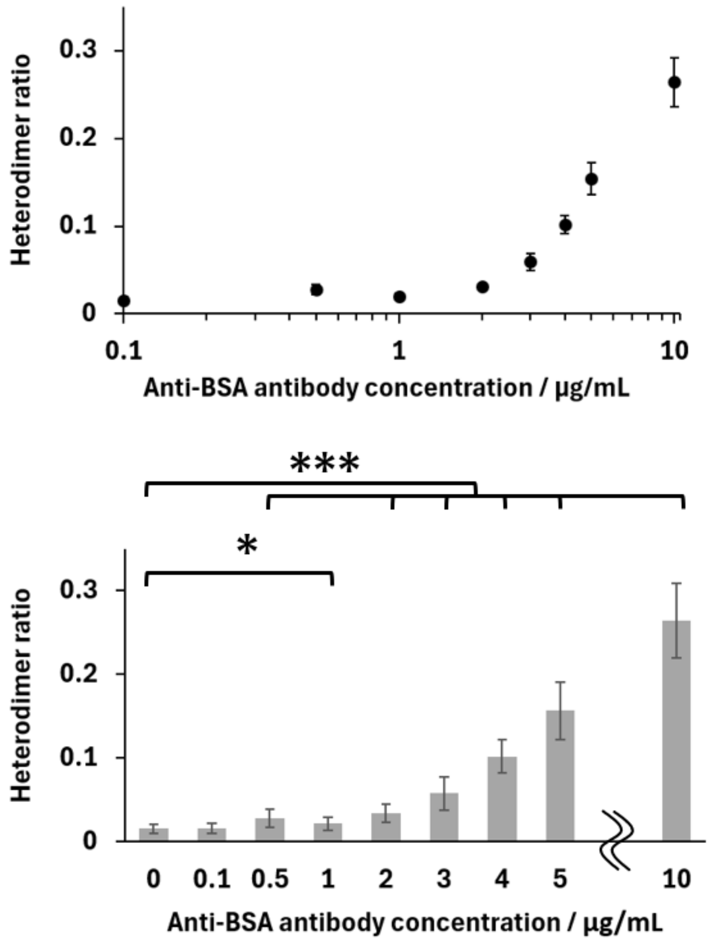

Figure S8 Anti-BSA antibody detection using heterodimer ratio based on spot classification by SVM on a logarithmic scale (upper) and a broken scale (lower). * p-value < 0.05; *** p-value < 0.005

Table S3 Spot classification by SVM algorithm

	AgNP-Protein A	AuNU-BSA	Heterodimer	Heterodimer ratio
0 µg/mL	719	4080	80	0.0155
1 µg/mL	810	2453	72	0.0200
2 µg/mL	776	2249	161	0.0312
10 µg/mL	164	422	211	0.264

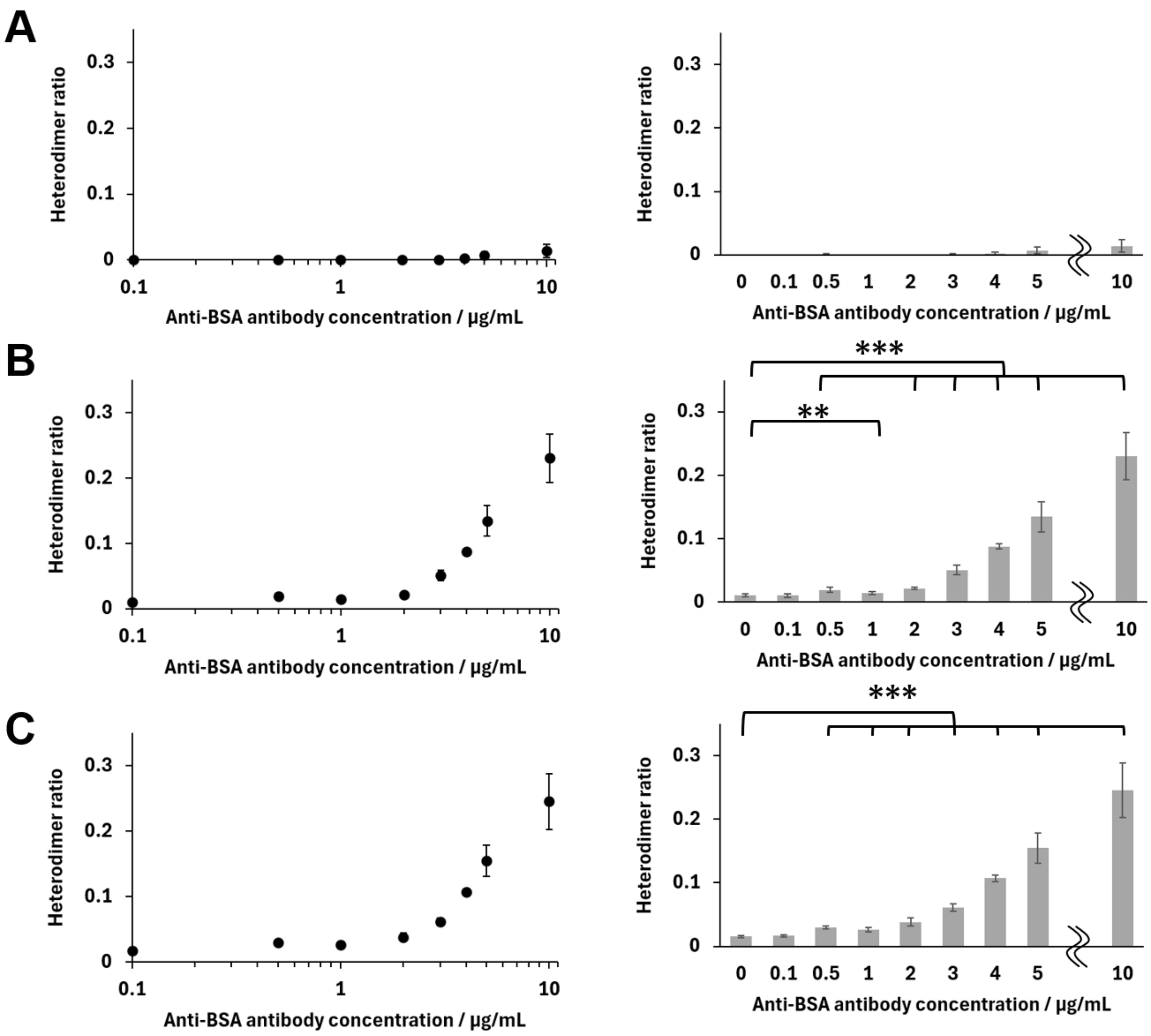

Figure S9 Anti-BSA antibody detection using heterodimer ratio based on spot classification by other machine learning methods using LDA (A), LR (B) and DT (C) on a logarithmic scale (left) and a broken scale (right). Machine learning analyses were conducted using R software with the "MASS", "stats" and "rpart" packages for LDA, LR and DT. The LOD values were N.D. (LDA) and 0.5 µg/mL (LR and DT), respectively. ** p-value < 0.01; *** p-value < 0.005

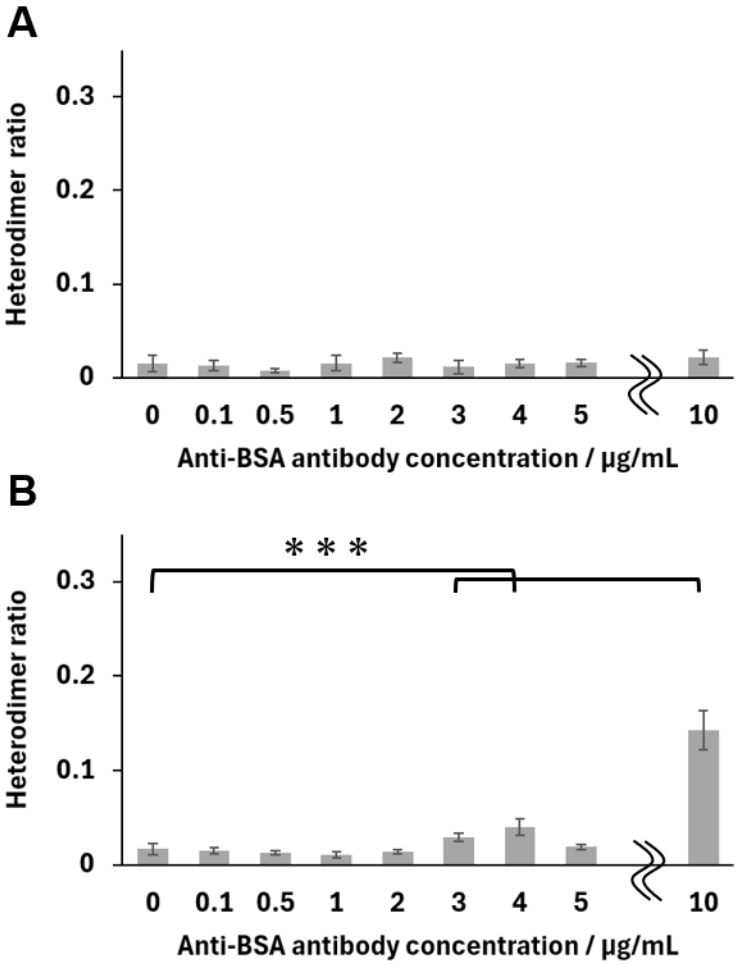

Figure S10 Heterodimer ratio using non-machine learning methods based on intensity (A) and area (B) information. *** p-value < 0.005
